# Supplementary material for: A Real-World Cost-Effectiveness Analysis of Rivaroxaban versus Vitamin K Antagonists for the Treatment of Symptomatic Venous Thromboembolism: Lessons from the REMOTEV Registry
Source: Medicina (Kaunas). 2023 Jan 16;59(1):181. doi: 10.3390/medicina59010181 (PMC9867052; doi:10.3390/medicina59010181)
Supplement: Supplementary file 1 [file medicina-59-00181-s001.zip › medicina-2127126-supplementary.pdf]

Table S1. Unit costs of resources use in euros.

		Unit Cost
Direct healthcare costs (national estimates)		
Biology		€1.89
Creatinine		€2.43
Aminotransferases		€4.05
Platelet count		€5.4
International Normalized Ratio		
Medics		€58.25
Rivaroxaban (30 tabs)	VKAs (30 tabs)	€3.93
Low Molecular Weight Heparin		€5.7
Fondaparinux		€5.1
Nurses		
Anticoagulant injection	Blood test	€7
		€8.58
Hospital admissions (French diagnosis related group)		
Pulmonary embolism		€4705
Deep vein thrombosis		€2494
Peripheral vascular disorder		€2855

Tabs: tablets; VKAs: vitamin K antagonists.
Weighted average for all four severity levels
